# Effect of vitamin E supplementation on chicken sperm quality: A meta-analysis

**DOI:** 10.14202/vetworld.2022.419-426

**Published:** 2022-02-24

**Authors:** Sari Yanti Hayanti, Cecep Hidayat, Anuraga Jayanegara, Mohammad Miftakhus Sholikin, Supardi Rusdiana, Yeni Widyaningrum, Masito Masito, Yenni Yusriani, Novia Qomariyah, Yenny Nur Anggraeny

**Affiliations:** 1 Jambi Assessment Institute for Agricultural Technology, Jambi City 36128, Indonesia; 2 Indonesian Research Institute for Animal Production, Ciawi, Bogor 16720, Indonesia; 3 Animal Feed and Nutrition Modelling Research Group, Faculty of Animal Science, IPB University, Bogor 16680, Indonesia; 4 Department of Nutrition and Feed Technology, Faculty of Animal Science, IPB University, Bogor 16680, Indonesia; 5 National Research and Innovation Agency of Indonesia, Jakarta 10340, Indonesia; 6 Beef Cattle Research Station, Pasuruan, East Java 67184, Indonesia; 7 South Sumatra Assessment Institute for Agricultural Technology, Palembang 30151, Indonesia; 8 Aceh Assessment Institute for Agricultural Technology, Banda Aceh 23125, Indonesia; 9 South Sulawesi Assessment Institute for Agricultural Technology, Makassar 90243, Indonesia

**Keywords:** meta-analysis, rooster, sperm, vitamin E

## Abstract

**Background and Aim:**

Among several factors, the sperm quality of poultry is affected by the rooster’s body size and the availability of antioxidants like vitamin E. This study aimed to determine the effect of dietary vitamin E supplementation on rooster sperm quality through a meta-analysis.

**Materials and Methods:**

After verification and evaluation, a total of 19 articles were included in this study. Data, including dietary vitamin E, semen volume, concentration, total sperm cells, pH, motility, viability, percentage of dead and abnormal sperm, vitamin E sperm content, malondialdehyde (MDA) content, and testosterone levels, were tabulated in a database; these were subsequently analyzed using mixed modeling with vitamin E dose as a fixed effect and study identity as a random effect.

**Results:**

Dietary supplementation level of vitamin E significantly (p<0.001) affected sperm concentration, significantly affected motility (p<0.001), significantly affected sperm vitamin E (p<0.001), significantly affected viability (p<0.001), and significantly affected chicken sperm fertility (p=0.001). Vitamin E administration also significantly reduced the number of sperm cell deaths (p<0.001); however, increased dietary levels of vitamin E did not affect semen volume (p=0.853), pH (p=0.951), MDA (p=0.542), the percentage of abnormal sperm cells (p=0.343), nor testosterone levels (p=0.063).

**Conclusion:**

Dietary vitamin E supplementation is recommended for male chickens since it generally enhances the quality of their sperm.

## Introduction

Low damage and death rates of sperm cells are important to ensuring the quality of healthy chicken sperm [1]. Cells typically have natural defense mechanisms to prevent free radical damage; however, if the concentration of antioxidants in seminal plasma decreases, the concentration of free-formed radical substances increases [2]. The chicken sperm cell membrane has a high concentration of polyunsaturated fatty acids, which increases cell activity through lipid peroxide processing to increase the reactive oxygen species (ROS) reaction [3]. Chicken sperm cells are also affected by low cytoplasm, which reduces the amount of natural antioxidants in the cells; if ROS increase, chicken sperm cells are more vulnerable to damage than those of other animals [4]. For decades, scientists have investigated the most effective methods of reducing harmful processes in both *in vivo* and *in vitro* chicken sperm [5]; one such method is to increase the amounts of antioxidants in *in vivo* sperm cells.

In chickens, increasing total antioxidants can improve sperm quality [6]. Since vitamin E was discovered in mice in the 1920s [7], poultry research has continued to progress with respect to investigating the effects of vitamin E as an antioxidant. On the cellular level, fat-soluble vitamin E protects cells from the damage caused by lipid peroxidase process ROS [8]. Although studies have demonstrated intracellular ROS reduction with vitamin E [9] in humans, this has never been done intracellularly in animal sperm, particularly poultry sperm. Vitamin E is an antioxidant with many protective functions, which have a positive effect on the improvement of rooster growth and resistance [10], and reproductive hormones [11] and in hens [12]. Meanwhile, introducing vitamin E to chicken sperm cells increases fertility to improve the reproductive performance of roosters [13].

Numerous studies of vitamin E supplementation in roosters have revealed several differentiating factors, including the chickens’ age and breed, as well as the vitamin E dose. Altogether, these three variables may play a role in determining the quality of chicken sperm [14-16]. As a result, a systematic review is required to determine the linearity of the effect of vitamin E on rooster sperm quality. Meta-analysis is a powerful technique for analyzing numerous studies conducted using consistent variables [17].

Therefore, the purpose of this study was to determine the effect of vitamin E supplementation on rooster sperm quality through a meta-analysis of previously published research.

## Materials and Methods

### Ethical approval

This is a meta-analysis, so ethical approval is not necessary for this study.

### Database development

This meta-analysis study follows the method in the Preferred Reporting Items for Systematic Reviews and Meta-Analyses guidelines reported by Selcuk [18]. All the data used in the present study were collected from published articles, which were then recorded in a database. Published articles were found using the keywords “vitamin E,” “ɑ-tocopherol,” “rooster sperm,” “cockerel sperm,” and/or “chicken sperm” to browse multiple search engines for scientific articles (i.e., Google Scholar, Scopus, Science Web, PubMed, and Mendeley).

### Inclusion/exclusion criteria

The main inclusion criteria were: (1) An English language journal article published by a reputable publisher; (2) the experimental design must have complied with the correct statistical rules; (3) the amount of experimental and replicated material met the correct statistical standards; (4) animal experimental material was used specifically for chickens; (5) vitamin E dose was supplied in mg/kg or could be converted to mg/kg; and (6) the number of chickens used (n) must have met the correct statistical requirements.

### Data extraction

From the selected articles, the following data were inputted: Author’s name(s), publication year, journal name, breedof chicken rooster, number of roosters, vitamin E treatment dose, observed parameters, recommended dose, source of vitamin E, units for each parameter, sampling technique, and parameter measurement technique.

Approximately 60 papers describing studies of vitamin E supplementation for roosters were initially retrieved, but only 40 of these papers had the potential to be included based on their title and abstract. The included parameters were semen volume, sperm concentration, total sperm, pH, motility, viability, percentage dead sperm, percentage abnormal sperm, fertility, sperm vitamin E, malondialdehyde (MDA) level, and testosterone level.

**Figure-1:**
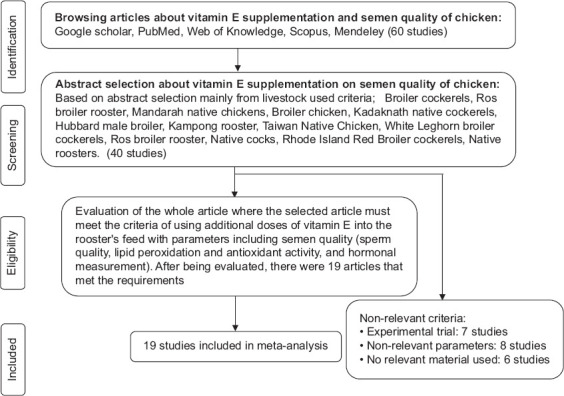
The selection of articles and evaluation process using PRISMA method. PRISMA=Preferred Reporting Items for Systematic Reviews and Meta-Analyses.

After a thorough assessment, 19 articles were selected for inclusion in the database (Table-1). The selected papers included eight studies with local breed roosters and 11 studies with broilers [13,19-36]. The roosters were between 18 and 65 weeks old. They were usually fed using factory produced feed with vitamin E supplementation at 0-13,400 mg/kg; whenever another dosing unit was used, it was converted to mg/kg for unit consistency. Vitamin E performance parameters included volume (mL), concentration (×10^9^/mL), total sperm cell (×10^9^/Ejac) pH, motility (%), viability (%), dead sperm (%), abnormal sperm (%), fertility (%), sperm vitamin E (ng/109 cell), MDA (nmol/mL), and testosterone (nmol/L). Dose and performance parameters for vitamin E were then entered into the database. For articles that used a different unit (e.g., ×10^9^/mL or ×10^9^/Ejac), data were transformed into a similar unit of measurement. Once all data regarding dietary vitamin E doses and semen performance were entered, the database was statistically analyzed. The article selection and evaluation process are visualized in Figure-1.

**Table 1:** Studies included in the meta-analysis.

S. No.	Reference	Chicken type	Age (week)	Number of animals	Parameters that were examined	Vitamin E dosage (mg/kg)	Dosage recommended (mg/kg)
1.	[13]	Broiler roosters	45	24	Volume, concentration, viability, MDA, fertility, testosterone,	30 and 200	200
2.	[19]	Broiler cockerels	32, 42, and 52	48	Volume, concentration, motility, sperm vitamin E	30 and 200	200
3.	[20]	Ross broiler rooster	30	24	Motility, viability, and MDA	30 and 200	200
4.	[21]	Mandarah native chickens	32	45	Volume, concentration, pH, motility, viability, fertility, total sperm cell	20.5 and 150	150
5.	[22]	Broiler chicken	22-52	32	Volume, concentration, motility, abnormal sperm, total sperm cell	100, 200, and 300	200
6.	[23]	Kadaknath native cockerels	30	135	Volume, concentration, motility, sperm vitamin E, viability, dead, abnormal, fertility	10, 100, and 200	100
7.	[24]	Mandarah native roosters	32-52	54	Volume, concentration, pH, motility, viability, fertility, total cell sperm	67 and 150	150
8.	[25]	Kampong rooster	60-64	45	Volume, concentration, motility, viability, dead, and abnormal cell sperm	0, 134, and 268	268
9.	[26]	Taiwan native chicken	23-52	90	Concentration, viability, fertility	0, 20, 40, 80, and 160	20-160
10.	[27]	Hubbard male broiler	65	180	Volume, concentration, motility, dead	33.5 and 67	67
11.	[28]	White Leghorn broiler cockerels	38-53	320	Volume, motility	20, 200, and 400	400
12.	[29]	Ros broiler rooster	45	36	Volume, concentration, motility, viability, MDA, fertility, testosterone, total cell sperm	30 and 200	200
13.	[30]	Native cocks	28	60	Volume, pH, abnormal, total sperm cell	0, 67, 670, 6700, and 13,400	67
14.	[31]	Rhode Island Red Broiler cockerels	24	60	Sperm vitamin E	0, 20, 200, and 1000	200
15.	[32]	Native roosters	45	120	Motility, viability, MDA, testosterone	0 and 200	200
16.	[33]	Mandarah native chickens	40	36	Volume, dead, total sperm cell	0 and 200	200
17.	[34]	Broiler roosters	45	30	Volume, concentration, motility, viability, MDA	0 and 200	200
18.	[35]	Broiler chickens	18	50	volume, concentration, motility, viability	45, 145, and 245	245
19.	[36]	Broiler male	22-54	64	Fertility	30 and 120	120

MDA=Malondialdehyde

### Statistical analysis

Data were processed using a mixed model procedure [37-39]. The analysis was performed using the PROC MIXED procedure in SAS version 9.1 (SAS Institute Inc., Cary, NC, USA) [40]. The vitamin E dose was defined as a fixed effect, whereas different studies were determined as random effects (declared in the RANDOM statement). The following mathematical model was employed:


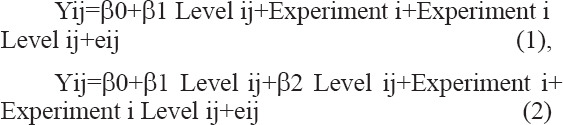


Where: (1) Linear mixed model (LMM) mathematical model in the 1^st^ order, (2) LMM mathematical model in the 2^nd^ order, β0+β1 Levelij (1^st^ order) and β0+β1 Level ij+β2 Level ij (2^nd^ order)=fixed effect, Experiment i+Experiment i Level ij (1^st^ and 2^nd^ order), β0 – overall intercept value across all experiments, β1 – linear regression coefficient of the 1^st^ order, β2 – linear regression coefficient of the 2^nd^ order, Level ij – additional level on the random effect, experiment – experiment i, and eij – unexplained residual errors. A linear regression model was applied when the respective quadratic regression model was not significant at p<0.05. Model statistics used were p-value and root mean square error. The significance of an effect was considered when p<0.05 [40].

For significant parameters with quadratic regression, the optimum dose of vitamin E can be determined to achieve the maximum performance of these parameters. The optimum dose of vitamin E can be obtained using the differential method of the quadratic regression equation, with the formula below.

Y=aX2+bx+c

dy/dx=2ax+b=0

2ax+b=0

X=−b/2a

X=optimum dose of vitamin E

## Results

### Sperm quality

The relationship between vitamin E dose and the sperm quality parameters is presented in Table-2. Vitamin E dose had a significant positive relationship (p<0.001) with sperm concentration significantly affected motility (p<0.001), viability (p<0.001), and fertility (p<0.001). On the other hand, the vitamin E dose significantly (p<0.001) decreased the mortality percentage. please change the sentence to “Meanwhile, the dose of vitamin E did not affect volume (p=0.853), pH (p=0.951), and percentage abnormal sperm cells (p=0.343).

**Table 2:** The effects of dosage vitamin E on semen quality of chicken.

Parameter	Unit	Model	N	Intercept	SE intercept	Slope	SE slope	p-value	RMSE	AIC	Trend
Volume	mL	L	51	0.358	0.047	−0.0000002	0.0000011	0.853	1.026	−71.1	Negative
pH		L	10	7.42	0.055	0.0000005	0.0000078	0.951	0.847	20.3	Positive
Concentration	10^9^/mL	Q	63	2.75	0.226	0.0013	0.00000023	<0.001			Positive
						0.0000015	0.00000001	<0.001	1.67	156	
Motility	%	L	54	60.9	5.05	0.0236	0.0042	<0.001	1.11	418	Positive
Viability	%	Q	66	64.8	4.04	0.114	0.0289	<0.001			Positive
						−0.00034	0.00011	0.004	1.17	570	
Dead	%	L	16	22.1	3.45	−0.0467	0.0027	<0.001	0.992	120	Negative
Abnormal	%	L	20	7.70	1.33	0.00019	0.000197	0.343	0.963	119	Positive
Fertility	%	L	38	71.9	4.80	0.011	0.0031	0.001	1.26	262	Positive
Sperm vitamin E	ng/10^9^ cells	Q	10	92.4	18.7	0.590	0.0063	<0.001			Positive
						−0.00049	0.000006	<0.001	0.786	104	
MDA	nmol/mL	L	22	2.89	0.253	0.00031	0.00049	0.542	0.918	66.5	Positive
Testosterone	nmol/L	L	12	2.61	0.137	−0.00058	0.00024	0.063	0.871	27.6	Negative

N=Sample size, RMSE=Root means square error, AIC=Akaike information criterion, MDA=Malondialdehyde,

SE=Standard error

### Lipid peroxidation and antioxidant activity

The effect of the vitamin E dose on lipid peroxidation and antioxidant activity is presented in Table-2. The dose of vitamin E significantly (p<0.001) increased the concentration of vitamin E in sperm; however, it did not affect (p=0.542) the MDA content in semen.

### Hormonal measurement

The effect of the vitamin E dose on testosterone level is shown in Table-2. The dose of vitamin E did not affect (p=0.063) the testosterone concentration.

## Discussion

Cerolini *et al*. [22] noted that vitamin E is a natural antioxidant that can improve semen quality and fertility. Biswas *et al*. [41] added that vitamin E protects spermatozoa from free radicals and lipid peroxidation, thereby helping to maintain optimal fertilization ability. Furthermore, Tabatabaei *et al*. [42] showed that vitamin E is a natural antioxidant that can improve semen quality and fertilization ability in chickens. Vitamin E has been widely used in poultry feed to increase the production and reproductive performance of poultry species [43]. Bréque *et al*. [44] demonstrated that dietary vitamin E supplementation effectively inhibits lipid peroxidation of the plasma membrane of chicken spermatozoa. Moreover, Asrol and Baba [25] reported that the semen quality of roosters (i.e., motility, percentage of spermatozoa viability, and cement color) increases with the dietary supplementation of 400 IU vitamin E for 4 weeks. In addition, vitamin E can also be used as a source of antioxidants in both the feed and drinking water for roosters. In general, all parameters differ at least 2-fold with vitamin E supplementation. Variations in parameters such as viability, motility, and fertility can be caused by differences in the breed [15] and the rooster’s age [45]. The results of Mavi *et al*. [15] and Lagares *et al*. [45] study correspond to those of the current meta-analysis, which showed the effect of vitamin E supplementation on rooster local breed chickens and broiler chickens aged between 18 and 65 weeks.

Like in male mammals, the male avian group has almost no accessory glands; this leads poultry to have a small amount of seminal plasma, leading to a low semen volume [46]. A recent study found that the volume of chicken semen can only reach a maximum of 0.9 mL [47]. In contrast, the maximum noted volume of chicken semen was only 0.8 mL in studies conducted some decades ago [48]. The results of the study by Perry [48] correspond to the descriptive results of this study, which demonstrated a maximum chicken semen volume of 0.8 mL; this is also consistent with the results of the current meta-analysis, which demonstrated that vitamin E does not affect chicken semen volume (Table-2). Moreover, in the current meta-analysis, the pH of chicken semen was similar with and without vitamin E supplementation. The pH of semen can change from the male to the female reproductive organs [49], and semen pH affects chicken sperm speed and motility [50].

Further research has shown that, in quail semen, acidic conditions (pH<6) cause sperm flagellum to become inactive [51]. Moreover, bull sperm cells move at maximum motility at pH 7.5, compared to pH 7 and 8 immediately following ejaculation [52]. Altogether, these results demonstrate that vitamin E supplementation in chickens can aid in maintaining a stable semen pH, such that sperm motility can increase or be preserved.

The success of the spermatogenesis process strongly influences sperm density during each ejaculation. The older the chicken, the lower the sperm organ’s production performance, which reduces the sperm cell density [45]. Cells in the testes, including Sertoli cells, Leydig cells, spermatogonia, and spermatids, may experience cell programmed death, such that cell organelles like the endoplasmatic reticulum may also be destroyed [53-55]. External (e.g., heat stress) and internal factors (e.g., sperm cell lipid content) may be responsible for a reduction in testicular weight and chicken spermatids due to ROS [3,56,57]. Vitamin E eliminates the effects of heat stress on chickentestes and sperm [58], preventing excess sperm cell death, and improving testicular volume density [6,14]. The testes have Sertoli cells, which maintain the cells until spermatogenesis is complete [59]. Vitamin E has an important part in the spermatogenesis process of chicken sperm cells, even at older ages [26]; this could have had a significant effect on the concentrations of chicken sperm noted in this meta-analysis due to the administration of vitamin E (Table-2).

Since it can improve chicken sperm concentration, vitamin E positively correlates with semen microscopic qualities, such as increasing motility and the viability percentage and suppressing the sperm death percentage. The increased concentration of ROS due to increased sperm cell metabolism induces adenosine triphosphate (ATP) production to inhibit sperm cell motility [60,61]. ROS also causes damage to the deoxyribonucleic acid (DNA) of sperm cell organelles and decreases sperm motility [62], and leads to sperm cell death after ejaculation [63].

The number of dead sperm significantly affected the sperm viability percentage. This meta-analysis illustrated the significant impact of vitamin E dose on motility, viability, and reducing sperm mortality (Table-2). According to our calculation, the optimal dose of vitamin E in terms of maximum viability was 166 mg/kg. Non-enzymatic antioxidant classified vitamin E breaks the chain in peroxidation reactions [64]. Several recent studies have illustrated a correlation between higher sperm motility and feasibility at lower ROS levels across different antioxidant types [65-67]. Moreover, improving the microscopic quality of sperm cells through vitamin E supplementation has an impact on fertilization. The vitamin E level significantly impacted the fertility of roosters through the high number of normal and mobile sperm. This result is in line with those of a study conducted by Cerolini *et al*. [22], who showed that vitamin E could prevent a decrease in infertility when subjected to thermal stress.

This meta-analysis revealed that the dose of vitamin E did not significantly affect MDA concentration (Table-2). According to our calculations, the optimum vitamin E dose to achieve the best results with respect to MDA was 101 mg/kg. This illustrates that the concentration of MDA is not the main cause of damage to sperm cells, which can decrease mobility percentage and viability and increase the rate of cell death. The previous studies stated that MDA is not a radical component and is not fully responsible for declines in avian sperm motility and fertility [31]. Moreover, recent study has shown that hydroxyl radicals and hydrogen peroxide hold most of the responsibility for decreased mitochondrial activity, DNA damage, increased lipid peroxide, and acrosomal, and plasma membrane disorders, by causing damage to the acrosome, DNA, plasma membrane, and mitochondria [68].

Nonetheless, generally, research on the impact of vitamin E on poultry semen has only investigated its effect on MDA. Furthermore, one study [68] showed that the concentration of MDA also increased due to the influence of the hydroxyl radical itself; this may explain why vitamin E does not directly reduce the concentration of MDA. Although further evidence is needed, the results of this meta-analysis suggest that improvement in the microscopic quality of chicken semen due to vitamin E supplementation suppresses the concentration of other radicals.

The supplementation of vitamin E to roosters was associated with an increase in the vitamin E concentration in sperm cells (Table-2). According to our calculations, the optimal dose for maximizing sperm vitamin E was 599 mg/kg. Adequate concentrations of vitamin E can increase vitamin E levels in all parts of the body [10], including sperm cells. With increased vitamin E concentrations in sperm cells, the cells in chicken semen do not experience oxidative stress [16], even after cryopreservation [69]; this causes the concentration of MDA and other radical components to rise, while the motility and viability percentage do not decrease and the mortality rate in sperm cells does not increase (Table-2). Increased vitamin E levels can also numerically control the number of abnormal sperm cells, although this was not a significant effect (Table-2).

Vitamin E can also play a role in preventing damage to Leydig cells. Under cultivation media, the administration of vitamin E to horse Leydig cells increases testosterone production [70]. Conversely, a study examining the effect of vitamin E *in vivo* in rats found no effect on testosterone levels [71]. Similarly, this meta-analysis, focusing on the effect of *in vivo* vitamin E in chickens, did not demonstrate an impact of vitamin E on testosterone levels (Table-2). Chen *et al*. [71] noted that this may be possible if the vitamin E concentration is not great enough to reach the steroidogenesis cells; however, this meta-analysis linked vitamin E dose to increased vitamin E levels in sperm cells, refuting this theory (Table-2). The high vitamin E levels in chicken sperm cells suggest that, since the spermatogenesis process is high, vitamin E may reach Leydig cells. Nonetheless, the reason for vitamin E has no effect on testosterone levels *in vivo* remains unknown.

## Conclusion

This meta-analysis demonstrated that vitamin E supplementation improves the quality of rooster sperm; thus, we recommend vitamin E supplementation in rooster feed as an important supplement. The recommended optimal dose of vitamin E supplementation in chicken feed is 599 mg/kg to optimize sperm vitamin E content, 166 mg/kg to maximize viability, and 101 mg/kg to optimize MDA content. The direction of future research development related to the use of vitamin E on reproductive performance in poultry should be directed at increasing the efficiency of the use of vitamin E doses through the application of nanotechnology, so that the dose of vitamin E can be further suppressed, and the effect can be further increased.

## Authors’ Contributions

SYH and CH: Conceptualized, designed, and wrote the original draft. MMS: Analyzed the data. SYH, YW, MM, and NQ: Searched the literature and collected and processed the data. CH, AJ, MMS, and NQ: Supervised the research. CH, AJ, SR, YY, and YNA: Critical review of the manuscript. All authors read and approved the final manuscript.
